# Colibactin in avian pathogenic *Escherichia coli* contributes to the development of meningitis in a mouse model

**DOI:** 10.1080/21505594.2021.1972538

**Published:** 2021-09-16

**Authors:** Peili Wang, Jiaxiang Zhang, Yanfei Chen, Haoran Zhong, Heng Wang, Jianji Li, Guoqiang Zhu, Pengpeng Xia, Luying Cui, Jun Li, Junsheng Dong, Qingqing Gao, Xia Meng

**Affiliations:** a College of Veterinary Medicine, Yangzhou University; Jiangsu Co-innovation Center for the Prevention and Control of Important Animal Infectious Diseases and Zoonoses, Yangzhou; b Joint International Research Laboratory of Agriculture and Agri-Product Safety, The Ministry of Education of China, Yangzhou

**Keywords:** Colibactin, meningitis, *Escherichia coli*, mouse model, *clbH*

## Abstract

Colibactin is synthesized by a 54-kb genomic island, leads to toxicity in eukaryotic cells, and plays a vital role in many diseases, including neonatal sepsis and meningitis. Avian pathogenic *Escherichia coli* (APEC) is speculated to be an armory of extraintestinal pathogenic *Escherichia coli* and can be a potential zoonotic bacterium that threatens human and animal health. In this study, the APEC XM meningitis mouse model was successfully established to investigate the effect of colibactin in *in vivo* infection. The *clbH*-deletion mutant strain induced lower γ-H2AX expression, no megalocytosis, and no cell cycle arrest in bEnd.3 cells, which showed that the deletion of *clbH* decreased the production of colibactin in the APEC XM strain. The deletion of *clbH* did not affect the APEC XM strain’s ability of adhering to and invading bEnd.3 cells. *In vitro*, the non-colibactin-producing strain displayed significantly lower serum resistance and it also induced a lower level of cytokine mRNA and few disruptions of tight junction proteins in infected bEnd.3 cells. Meningitis did not occur in APEC Δ*clbH*-infected mice *in vivo*, who showed fewer clinical symptoms and fewer lesions on radiological and histopathological analyses. Compared with the APEX XM strain, APEC Δ*clbH* induced lower bacterial colonization in tissues, lower mRNA expression of cytokines in brain tissues, and slight destruction of the brain blood barrier. These results indicate that *clbH* is a necessary component for the synthesis of genotoxic colibactin, and colibactin is related to the development of meningitis induced by APEC XM.

## Introduction

Colibactin is a natural and genotoxic chemical compound that was first detected and identified in a neonatal meningitis *Escherichia coli* (NMEC) strain (IHE3034) by Nougayrède in 2006 [1]. It induces DNA double-strand breakage, chromosomal aberrations, and cell cycle arrest in the G2/M phase [1,2]. Colibactin is synthesized by a 54-kb genomic island (*pks* island), composed of 19 genes, by activation of the phosphopantetheinyl transferase (*clbA*), the cyclopropane-formatting synthetase (*clbH* and *clbI*) prodrug transporter (*clbM*), and colibactin-maturing peptidase (*clbP*). Numerous studies have shown that colibactin leads to severe toxicity in eukaryotic cells and plays an essential role in gut homeostasis [3], colorectal cancer [4], and neonatal sepsis/meningitis [5].

Avian pathogenic *Escherichia coli* (APEC), a principal member of the extraintestinal pathogenic *Escherichia coli* (ExPEC) group, induces severe respiratory and systemic diseases in poultry and leads to extensive economic losses. NMEC is another important member of the ExPEC group and is the most common secondary cause of central nervous system (CNS) infections in newborns with high morbidity and mortality [6]. Based on genotypic and phylogenetic group studies, both APEC and NMEC showed discernible phylogenetic overlaps and shared some virulence-associated factors, such as type 1 fimbriae, increased serum survival, and salmochelin [7,8]. Furthermore, several studies have demonstrated that APEC induces bacteremia or meningitis in neonatal rat or mouse models [9,10]. Therefore, APEC strains are speculated to be an armory of NMEC and can be potential zoonotic bacteria.

*E. coli* strains carrying the *pks* island can be isolated from multiple parts of the human body and confirm a natural transmission from mothers to their offspring [3]. The positive rates of *pks*^+^ *E. coli* isolated from the gut are relatively low in healthy adults (19.7% [11] to 32% [12]) and neonates (26.9% to 33%) [13]. However, the percentage of *E. coli* harboring *pks* island, increases distinctly in infectious disease isolates. *E. coli* is considered to be responsible for urosepsis [14], prostatitis [15], septicemia [12], and newborn meningitis [16]. The *pks* island is also strongly linked with *E. coli* strains of the phylogroup B2 [11,13] and with several virulence factors (adhesins, hemolysins, toxins, and siderophores) [11]. Interestingly, a majority of the NMEC (67.92% to 78.8%) belong to the B2 phylogenetic group [17,18]. Several virulence factors related to the *pks* island have also been shown to be involved in infant meningitis [19]. Therefore, the function of the *pks* island has been investigated in the etiology of neonatal meningitis, such as in colonization in the immature gut, translocation to the bloodstream [20], apoptosis of T lymphocytes, and development of septicemia [21]. Furthermore, *pks*^+^ *Klebsiella pneumoniae* has significant tropism toward the brain of BALB/c mice, and colibactin plays a key role in the pathogenic steps that lead to the development of meningitis [5]. However, the function of colibactin in the pathogenesis of *E. coli* meningitis is still unclear. As mature colibactin is still difficult to extract in the purified form from bacteria to date [22], it is difficult to investigate the role of colibactin in the development of meningitis. *clbH* belongs to the *pks* island and is involved in the formation of the genotoxic necessary AM-ACP unit [23]. Therefore, we selected *clbH* to block the synthesis of colibactin in the APEC XM strain. The *E. coli* meningitis mouse model was established to evaluate the role of colibactin in the development of meningitis in this study.

## Method and material

### Ethics statement

The animal experiments followed the National Institute of Health guidelines for the ethical use of animals in China. All procedures were approved by the Animal Care and Ethics Committee of Yangzhou University. Four-week-old Institute of Cancer Research (ICR) mice were provided by the Comparative Medicine Center of Yangzhou University (License number: SCXK (Su) 2017–0007) and had free access to food and water under a 12 h light/dark cycle with observation twice a day. All manipulations were performed under anesthesia to minimize the suffering of animals. The mice were euthanized with overdose isoflurane exposure and samples were collected for analysis.

### Strains, growth conditions and plasmids

The APEC XM strain (O2:K1) was isolated from the brain of a duck with symptoms of septicemia and meningitis (donated by Dr. Guoqiang Zhu, Yangzhou University). It grew aerobically on Luria-Bertani (LB) plates or in LB broth with agitation (180 rpm) at 37°C. When necessary, antibiotics were added with the following concentrations: ampicillin (100 μg/mL) or chloramphenicol (34 μg/mL). The strains and plasmids used in this study are listed in Table 1.

**Table 1.** Summary of bacterial strains, plasmids, and primers used in this study

Strain or plasmid	Characteristic or function	Source
Strains		
APEC XM	Virulent strain of APEC	Donated by Dr. Guoqiang Zhu
APEC Δ*clbH*	Deletion mutant of *clbH* with APEC XM background	This study
APEC Δ*clbH*/p*clbH*	APEC Δ*clbH* with the vector pBR322-*clbH*, Amp^r^	This study
Plasmid		
pKD46	λ red recombinase expression plasmid	Collected in our lab
pKD3	pANTSγ derivative containing FRT-flanked, Cm^r^	Collected in our lab
pCP20	temperature-sensitive replication and thermal induction of FLP synthesis	Collected in our lab
pBR322-*clbH*	pBR322 containing the promoter followed by the full-length *clbH*, Amp^r^	This study
Primer	Sequence (5ʹ→3ʹ)	Product size
P1	CGATTGAGCCGATGACAAC	1560/500
P2	ACAGCAAGGGATTATGAGACA	
P3	CGCATCGCATGGGCTTCGATTTCTCCCAATTCAACGCGCAACCCTCTTGTGTAGGCTGGAGCTGCTTCG	1360
P4	CTACCGACGACCAAACGATAGTCGACTATTTGTATCGTATCGCCGGAGCATATGAATATCCTCCTTAG	
P5	TAACGCAGTCAGGCACCGTGTATGGAACAGCAAGGGATTATGAG	1560
P6	GTGAATCCGTTAGCGAGGTGCCTCAGTGATGACTGTCGGTTGTG	
*GAPDH*	AACGGGAAGCCCATCACCATC	98
	AAGACACCAGTAGACTCCACGA	
*IL-1β*	ATGAAAGACGGCACACCCAC	175
	GCTTGTGCTCTGCTTGTGAG	
*IL-6*	TGCAAGAGACTTCCATCCAGT	71
	GTGAAGTAGGGAAGGCCG	
*TNF-α*	ACTGAACTTCGGGGTGATCG	97
	TGATCTGAGTGTGAGGGTCTGG	

### *Construction of the* clbH *deletion mutant and complemented mutant*

The deletion of *clbH* in the chromosome of APEC XM strain was achieved using bacteriophage λ Red recombinase system with primers and plasmid pKD3, pKD46, and pCP20 as described previously [24] (Table 1). For the construction of complemented mutant, the coding sequences of *clbH* gene were amplified from the APEC XM genome and cloned into plasmid pBR322. Polymerase Chain Reaction (PCR) and DNA sequencing confirmed modified genotypes in mutant strains. All primers used in this study are listed in Table 1.

### Growth curves

Briefly, 200 μL (1 × 10^8^ CFU/mL) bacteria in the exponential phase were inoculated in 20 mL LB medium with or without ampicillin in a 37°C shaking incubator at 180 rpm for 22 hours. The absorbance of bacterial culture was recorded per hour by spectrophotometer at 630 nm. The above experiments were repeated independently three times. The growth curves were drawn by GraphPad Prism 5.0 software (GraphPad Software).

### Colibactin cytotoxicity assays

In the present study, bEnd.3 cells (American Type Culture Collection, ATCC CRL-2299) were used to demonstrate the cytotoxic effect of colibactin on eukaryotic cells. The cells were cultured in the DMEM (Gibco, 12800-017), supplemented with 10% heat-inactivated fetal bovine serum (FBS; Gibco, 16140-071) at 37°C in a humidified 5% CO_2_ atmosphere. The bEnd.3 cells (about 75% confluence) were infected with bacteria in the exponential phase with a multiplicity of infection (MOI) of 100. After 4 h infection, the cells were washed three times with PBS, and further incubated in DMEM with 10% FBS containing gentamicin (100 μg/mL) for the following analysis.

The bEnd.3 cells were observed for megalocytosis at 72 hours post-incubation (hpi) [25]. The cells were fixed with 4% paraformaldehyde for 20 min, and then stained with 0.1% methylene blue for 20 min. The megalocytosis of cells were observed by an inverted microscope. Cytotoxic effects of colibactin produced by APEC XM, APEC Δ*clbH*, or APEC Δ*clbH*/p*clbH* were quantified by measurement of absorbance at 630 nm using a microplate reader. The expression of γ-H2AX in bEnd.3 cells were detected at 0 and 72 hpi. After washing 3 times, the cells were fixed with 4% paraformaldehyde for 20 min, permeabilized with 0.1% Triton X-100 for 20 min and processed for immunofluorescence following a standard protocol [26]. The primary antibody was a monoclonal rabbit anti phosphorylated H2AX (Cell Signaling Technology, #9718). The secondary antibody was a goat-anti-rabbit IgG (H + L) Alexa Fluor Plus 488 (ThermoFisher Scientific, A-21070). Then, the cells were stained with 4ʹ, 6-diamidino-2-phenylindole (DAPI; Beyotime Biotechnology, C1002). Finally, the coverslips were fixed using a fluorescence mounting medium. The GFP fluorescence was detected and photographed by a fluorescence microscope (Leica, Germany).

Four hours after the infection, the cell cycle of bEnd.3 cells were measured at 48 hpi [1,25]. The cells were collected, centrifuged at 400 g for 5 min at 4°C, washed with PBS, and resuspended in 70% ice-old ethanol for fixation at 4°C overnight. The cells were then centrifuged at 800 g for 10 min at 4°C, washed with PBS, and stained with FxCycle™ PI/RNase staining solution (Thermo Fisher Scientific, F10797) at room temperature for 15 min. The cell cycle was monitored on the BD LSRFortessa flow cytometer (BD Biosciences, Franklin Lakes, NJ, USA) with 10,000 events/determination and analyzed with Flowjo software (Tree Star Inc.). The experiments were repeated three times independently.

### Bacterial resistance to normal mouse serum

Serum resistance assay was performed in a 96-well plate as described previously [27]. Briefly, specific-pathogen-free (SPF) mouse serum was diluted to 50% with PBS. APEC XM, APEC Δ*clbH*, and APEC Δ*clbH*/p*clbH* strain grown to exponential phase were collected and washed twice with PBS. A dose of 10 μL culture suspension (OD_600_ = 1.0) was inoculated into a 96-well plate containing 190 μL of 50% and 100% serum. After incubation for 0.5 h at 37°C, bacterial numbers were calculated using LB plates. The assay was performed in triplicate with three independent experiments.

### Adhesion and invasion assay

For adhesion and invasion assay, the strains were grown in LB medium with or without ampicillin in a 37°C shaking incubator at 180 rpm until the optical density at 600 nm reached 1.0 (1 × 10^8^ CFU/mL) in exponential phase. The bacteria were collected by centrifugation (3,500 rpm, 8 min), washed twice with phosphate-buffered saline (PBS), and resuspended in FBS-free DMEM. Then, bEnd.3 cells were infected with the APEC XM, APEC Δ*clbH*, or APEC Δ*clbH*/p*clbH* strain at a MOI of 100 for 4 h at 37°C in 5% CO_2_. The mock-infection cells were cultured in FBS-free DMEM as the control. The bEnd.3 cells were gently washed with PBS three times to remove any non-adherent bacteria, and then lysed with 0.5% Triton X-100 for 30 min at 37°C. The suspensions were collected, serially diluted 10-fold, and plated on LB plates. After incubation overnight at 37°C, the number of CFUs was calculated.

### *Relative mRNA expression of cytokines and tight junction proteins* in vivo *and* in vitro *infection with qRT-PCR*

*In vitro* infection, bEnd.3 cells were infected with the APEC XM, APEC Δ*clbH*, or APEC Δ*clbH*/p*clbH* strain at a MOI of 100 for 4 h at 37°C in 5% CO_2_. The mock-infection cells were cultured in FBS-free DMEM as control. The bEnd.3 cells were gently washed with PBS three times and the total RNA was extracted with TRIzol solution (Invitrogen, 15596-018). *In vivo* infection, the left hemisphere of brain was homogenized in TRIzol reagent and total RNA was extracted with TRIzol solution. The 900 ng of high-quality RNA was converted into cDNA by PrimeScript RT reagent Kit with gDNA Eraser (Takara, RR047A). qRT-PCR was performed on a CFX CONNECT Real-time PCR machine (Bio-Rad, CFX CONNECT, USA) using ChamQ SYBR qRT-PCR Master Mix (2×) (Vazyme, Q311-02) according to the manufacturer’s instructions. The amplification cycles were performed as follows: 95°C for 10 min, followed by 40 cycles of 95°C for 30 s, 60°C for 30 s, and 72°C for 30 s, and a final extension at 72°C for 10 minutes. The 2^−ΔΔCt^ method was used to analyze the gene expression. The primer sequences of cytokines and tight junction proteins are shown in Table 1.

**Table:** 

Primer	Sequence (5ʹ→3ʹ)	Product size
P1	CGATTGAGCCGATGACAAC	1560/500
P2	ACAGCAAGGGATTATGAGACA	
P3	CGCATCGCATGGGCTTCGATTTCTCCCAATTCAACGCGCAACCCTCTTGTGTAGGCTGGAGCTGCTTCG	1360
P4	CTACCGACGACCAAACGATAGTCGACTATTTGTATCGTATCGCCGGAGCATATGAATATCCTCCTTAG	
P5	TAACGCAGTCAGGCACCGTGTATGGAACAGCAAGGGATTATGAG	1560
P6	GTGAATCCGTTAGCGAGGTGCCTCAGTGATGACTGTCGGTTGTG	
*GAPDH*	AACGGGAAGCCCATCACCATC	98
	AAGACACCAGTAGACTCCACGA	
*IL-1β*	ATGAAAGACGGCACACCCAC	175
	GCTTGTGCTCTGCTTGTGAG	
*IL-6*	TGCAAGAGACTTCCATCCAGT	71
	GTGAAGTAGGGAAGGCCG	
*TNF-α*	ACTGAACTTCGGGGTGATCG	97
	TGATCTGAGTGTGAGGGTCTGG	

### *Expression of tight junction proteins examined* in vivo *and* in vitro *infection*

*In vitro* infection, bEnd.3 cells were infected with the APEC XM, APEC Δ*clbH*, or APEC Δ*clbH*/p*clbH* strain at a MOI of 100 for 4 h at 37°C in 5% CO_2_. The mock-infection cells were cultured in FBS-free DMEM as control. The bEnd.3 cells were gently washed with PBS three times and total proteins were extracted from bEnd.3 cells using RIPA Lysate Buffer (Beyotime Biotechnology, P0013B). *In vivo* infection, total proteins were extracted from the brains using RIPA Lysate Buffer. The concentrations were determined with a bicinchoninic acid protein assay kit (Beyotime Biotechnology, P0010). After SDS-PAGE separated the total proteins, the proteins were transferred to polyvinyl difluoride membranes (Millipore, ISEQ00010). The membranes were incubated with 5% skim milk for 1 h. And then, the membranes were cultured with primary antibodies overnight at 4°C, including ZO-1 (1:1000; Invitrogen, Cat#61-7300), occludin (1:500; Invitrogen, Cat#71-1500), claudin-5 (1:50; Invitrogen, Cat#35-2500), and GAPDH (1:1000; Cell Signaling Technology, Cat#2118). The membranes were washed with Tris-buffered saline/ Tween (TBS-T) buffer and incubated with horseradish peroxidase (HRP)-conjugated secondary antibodies (all at 1:10,000 dilution in 5% nonfat milk) at room temperature for 1 h. After washed with TBS-T, the membranes were incubated with enhanced chemiluminescence (Clinx Science Instruments, 1800212) for 30 s and detected by a chemiluminescence imaging system (Clinx Science Instruments, ChemiScope 5300, China). The band intensity was analyzed using a chemiluminescence imaging system (ChemiScope 5300; Clinx Science Instruments).

### *Construction of mouse meningitis model infected by* E. coli

Animal infection experiments were carried out to determine the infection rate and colonization ability in the brain, blood, and lung. Briefly, forty 4-week-old ICR mice were randomly divided into four groups. Each mouse was intraperitoneally injected with a dose of 10^7^ CFU in 100 μL normal saline or 100 μL sterilization normal saline [28]. After 8h post of infection (poi), clinical symptoms were observed per hour. Cerebrospinal fluid (CSF) samples were obtained by cisterna magna puncture with isoflurane inhalation anesthesia at 12 h poi. The whole blood samples were collected and treated with dipotassium ethylenediaminetetraacetic acid (K_2_-EDTA). Complete blood count test was performed using an automatic blood cell analyzer (Mindray, BC-1900, China). The brains tissues were collected, frozen Instantly in liquid nitrogen, and then stored at −80°C until used for detecting proinflammatory cytokines and tight junction proteins.

### Evans blue (EB) permeability assay

At 30 min before euthanasia, the mice were injected with 2% Evans blue solution (100 µL per mouse) into the caudal vein. Afterward, the mice were anesthetized and perfused with 50 mL of ice-cold PBS. Brain tissues were homogenized in 1100 µL pre-cool PBS, and then centrifuged at 15,000 g for 30 min at 4°C [29]. Each 500 µL supernatant was added with an equal amount of 50% trichloroacetic acid. After 12 h incubation at 4°C, the mixtures were centrifuged at 15,000 g for 30 min at 4°C to separate the supernatants. The absorbance was measured at 630 nm using a spectrophotometer.

### Bacterial loadings of blood, brain, lung, and CSF

The right hemisphere of the brain, lung, blood, and CSF samples were aseptically harvested and homogenized with sterile pre-cool PBS. After serial 10-fold dilutions in sterile PBS, 10 μL dilution was plated on MacConkey plates and cultured at 37°C. The bacterial loadings were calculated by CFU per gram of organs or per microliter of blood.

### Brain histopathology

At 12 h poi, the brains were carefully collected and immediately fixed in 4% paraformaldehyde. After 48 h, the tissues were dehydrated by serial gradient alcohol and xylene, and then embedded in paraffin. The embedded tissues were cut into 4 µm paraffin sections by an automated microtome (Leica, Germany) and stained with hematoxylin and eosin afterward. The brain sections were observed and analyzed by microscope (Nikon, Eclipse 80i, Japan).

### Immunohistochemistry detection of ZO-1, occludin, and claudin-5 proteins

The brain sections were prepared as mentioned above. The active endogenous peroxidase was blocked by 3% hydrogen peroxide. The sections were placed in the citrate buffer at 100°C for 15 min and then incubated with 5% bovine serum albumin (BSA; Boster Biological Technology, Cat#SA1020) at 37°C for 1 h. The following primary antibodies were used, including ZO-1 (1:100; Invitrogen, Cat#61-7300), occludin (1:100; Invitrogen, Cat#71-1500), and claudin-5 (1:200; Invitrogen, Cat#35-2500). After incubation with primary antibodies at 4°C overnight, the sections were incubated with secondary antibody, which was linked with HRP, and then stained with 0.1% 3, 3ʹ-diaminobenzidine (DAB; Boster Biological Technology, AR1000). The sections were dyed with hematoxylin and observed by a microscope (Leica, Germany). Images were analyzed by the soft Image J.

### Magnetic resonance imaging (MRI) scan

The MRI scanning was performed on a 7.0-T MRI scanner (Bruker Corporation, BRUKER BIOSPEC 70/30, Germany). The mice were anaesthetized by isoflurane inhalation. Then, the heads were fixed with two flat head plastic thumbscrews and the mice were placed on a heating pad for maintaining body temperature within 36.5°C to 37.5°C. The mouse was monitored during the scanning, including saturation of pulse oxygen, heart rate, respiratory rate, and rectal temperature. The MRI sequences used in this study were T1-weighted imaging and meglumine gadopentetate-enhanced T1-weighted imaging.

## Statistical analysis

Statistical analysis was performed using SPSS 16.0 (SPSS Inc.). Data showed as mean ± standard error of the mean from triplicate independent experiments. *P*-values were calculated using one-way ANOVA test. A *p*-value of less than 0.05 was considered statistically significant.

## Results

### clbH *has no effect on APEC growth*

**Figure 1.**
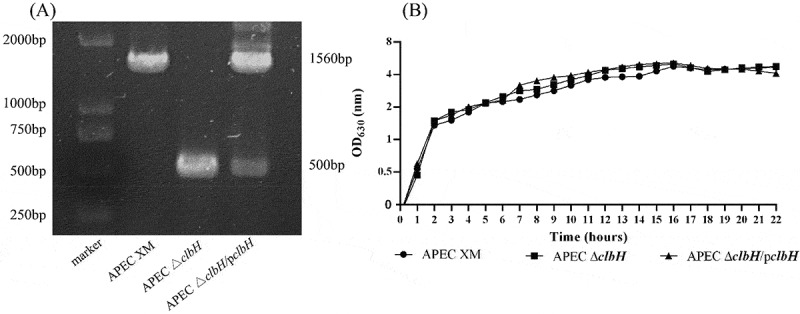
Verification of mutant strains using polymerized chain reaction (PCR) and testing their ability of reproduction

DNA sequencing and PCR (Figure 1 A) confirmed the deletion (APEC Δ*clbH)* and complemented (APEC Δ*clbH*/p*clbH)* strains. The DNA sequencing results also showed that the deletion and complemented strains were stable without any spurious mutations in the LB medium after 30 generations. As shown in Figure 1b, the growth curves of the APEC XM, APEC Δ*clbH*, and APEC Δ*clbH*/p*clbH* strains were recorded and depicted during the exponential growth and stationary phases. No significant differences were observed among the three strains. Therefore, *clbH* deletion and complemented strains were constructed, and *clbH* did not affect the ability of reproduction in APEC XM.

(A) Identification of mutant strains using PCR. The APEC XM, APEC Δ*clbH*, and APEC Δ*clbH*/p*clbH* strains were amplified using primers P1/P2. (B) Growth curves of the three strains, APEC XM, APEC Δ*clbH*, and APEC Δ*clbH*/p*clbH*, grown in LB broth at 37°C under aerobic conditions. The optical density (OD) of the culture was measured at OD_630_. Each curve was presented as the average of the three independent experiments. There were no differences among the three strains.

### clbH *is involved in colibactin production and elicits* in vitro *genotoxic effects*

**Figure 2.**
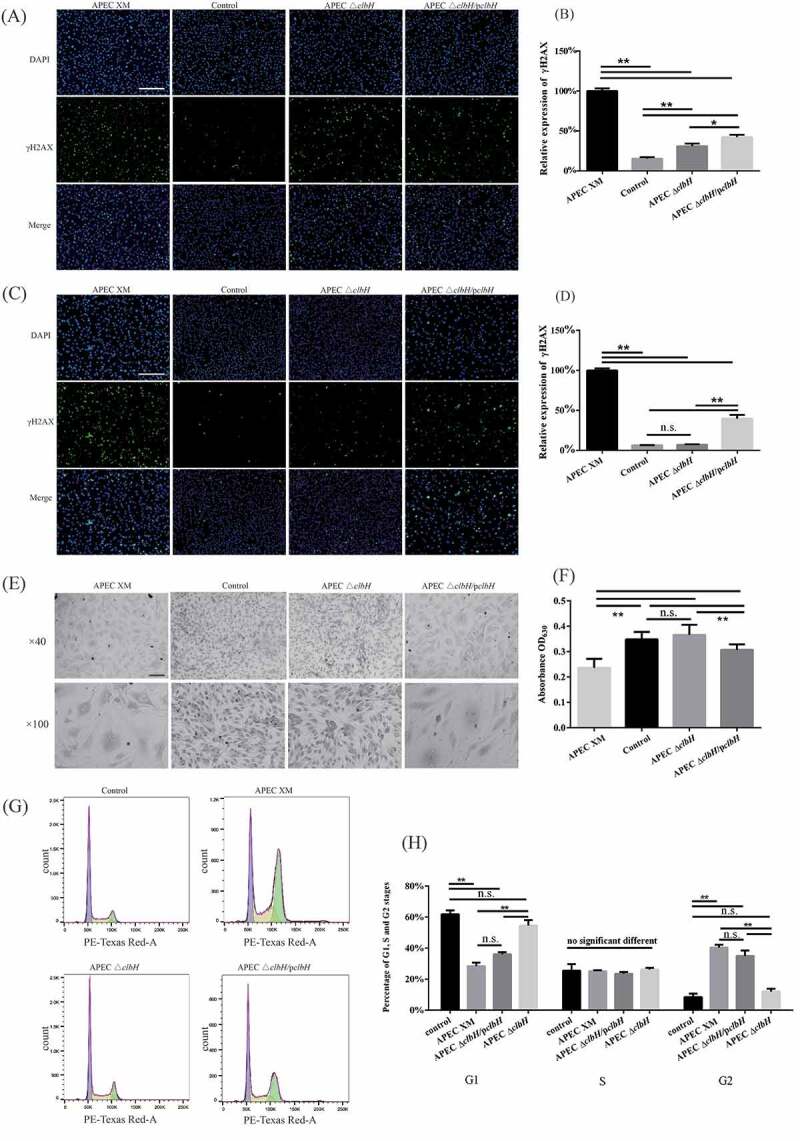
Colibactin production by APEC XM induces genotoxic effects in bEnd.3 cells

Cytotoxic effects of colibactin produced by the three strains were determined by quantification of H2AX phosphorylation, megalocytosis, and cell-cycle distribution. The percentage of γ-H2AX-positive cells was detected by an immunofluorescence assay as previously described [30]. The rate of increase in the γ-H2AX positive-cell numbers in the APEC XM group significantly increased at 0 and 72 hpi, compared to that in the control group. However, the expression of γ-H2AX in bEnd.3 cells infected with APEC Δ*clbH* was lower than that in the APEC XM group at 0 and 72 hpi (*p* < 0.01, Figure 2 A-D). Furthermore, there were no differences between the APEC Δ*clbH* and control groups at 72 hpi (*p* > 0.05; Figure 2 C, D). Additionally, the expression of γ-H2AX in the APEC Δ*clbH*/p*clbH* group was higher (*p* < 0.01; Figure 2 A-D) than that in the control group. Compared with that in the APEC XM group, the rate of γ-H2AX positive-cell numbers in the APEC Δ*clbH*/p*clbH* group significantly decreased (*p* < 0.01; Figure 2 A-D) at both time points indicating partial restoration of genotoxicity.

Bend.3 cells were infected for 4 h with APEC XM, APEC Δ*clbH*, or APEC Δ*clbH/*p*clbH* at 100 multiplicity of infection (MOI). For the control group, cells were not infected with any bacteria for 4 h. Variation and quantification of γ-H2AX expression in bEnd.3 cells at 0 (A, B) and at 72 hour post-incubation (hpi) (C, D). Nuclear DNA and γ-H2AX were colored in blue and green, respectively (bar = 200 μm). Data were reported as a percentage of γ-H2AX-positive cells in the overall cell population, and the rate of the APEC XM group was set as 100%. (E) Megalocytosis induced by colibactin was observed at 72 hpi by an inverted microscope (bar = 100 μm). (F) Quantification of megalocytosis was carried out by measuring the absorbance of methylene blue-stained cells at optical density (OD) of 600 nm. The data of the control group was set as 100%. (G) The cell cycles of bEnd.3 cells were assayed by flow cytometry (purple, G0/G1 phase; yellow, S phase; green, G2/M phase). APEC XM and APEC XM Δ*clbH/*p*clbH* resulted in G2/M phase accumulation in infected cells at 48 hpi. (H) Quantification analyses of the cell cycle analysis of bEnd.3 cells in each group after 48 hpi. The results were analyzed with one‐way ANOVA and presented as the mean ± standard errors of the mean for three independent experiments. (**, *p* < 0.01, *, 0.01 < *p* < 0.05, n.s., *p* > 0.05.)

Meanwhile, APEC XM induced megalocytosis in bEnd.3 cells, which was characterized by a progressive enlargement of the cell body and nucleus, and is quantified using methylene blue staining. APEC Δ*clbH* resulted in fewer giant cells and higher absorbance of staining (*p* < 0.01; Figure 2 E, F) than the APEC XM group. Furthermore, APEC Δ*clbH*/p*clbH* caused higher absorbance of methylene blue staining than that in the APEC XM group (*p* < 0.01; Figure 2 F) but lower than that in the control group (*p* < 0.01; Figure 2 F).

The cell cycle analysis showed a significant increase in the number of bEnd.3 cells in the G2 phase in the APEC XM and APEC Δ*clbH*/p*clbH* groups (*p* < 0.01; Figure 2 G, H), compared with that in the control group. In addition, the percentage of bEnd.3 cells in the G1 phase decreased significantly in the APEC XM (*p* < 0.01; Figure 2 G, H) and APEC Δ*clbH*/p*clbH* groups (*p* < 0.01; Figure 2 G, H) compared with that in the control group. Furthermore, there were no significant differences in the number of bEnd.3 cells in the G2 phase between the APEC Δ*clbH* and control groups (*p* > 0.05; Figure 2 G, H).

### Colibactin decreases serum resistance and does not contribute to the adhesion and invasion of bEnd.3 cells

**Figure 3.**
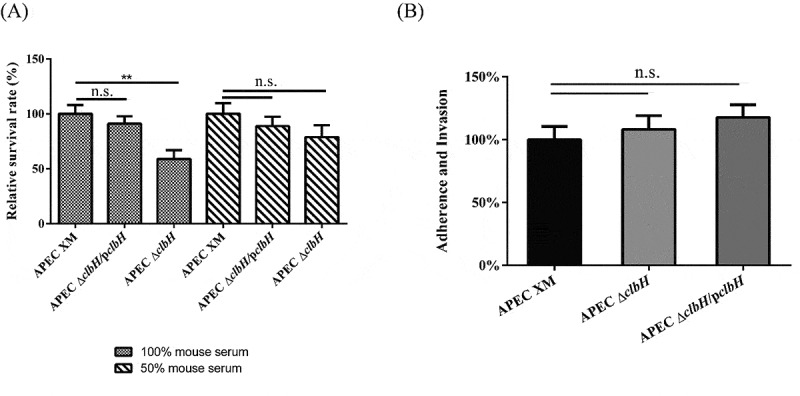
Serum resistance assay and adhesion and invasion assays

*E. coli* requires a high degree of bacteremia, and binding to and invasion of brain microvascular endothelial cells before it can traverse the blood brain barrier (BBB) [31–34]. The serum resistance assay showed that the APEC Δ*clbH* strain displayed significantly lower serum resistance than the APEC XM strain inoculated into 100% mouse serum (*p* < 0.01; Figure 3 A). In addition, the adhesion and invasion assays showed that there were no significant differences in the binding and invasion of bEnd.3 cells among the APEC Δ*clbH*, APEC Δ*clbH*/p*clbH*, and APEC XM groups (*p* > 0.05; Figure 3 B).

(A) For the serum resistance assay, the APEC XM, APEC Δ*clbH*, and APEC Δ*clbH*/p*clbH* strains were treated with 100% or 50% serum for 30 min, and bacterial numbers were calculated using LB plates. Data of the APEC XM were set as 100%. bEnd.3 cells were infected with a multiplicity of infection (MOI) of 100 bacteria/cell for 4 h in the APEC XM, APEC Δ*clbH*, or APEC Δ*clbH*/p*clbH* groups. For the control group, cells were not infected with any bacteria for 4 h. (B) For the adhesion and invasion assay, after 4 h of treatment and three washes with PBS, the bacteria were collected and analyzed using LB plates. The data of APEC XM was set as 100%. The data were analyzed with one‐way ANOVA and expressed as the mean ± standard error of the mean for three independent experiments. (**, *p* < 0.01, *, 0.01 < *p* < 0.05, n.s., *p* > 0.05.)

### *Relative mRNA cytokine expression in* in vitro *infection*

**Figure 4.**
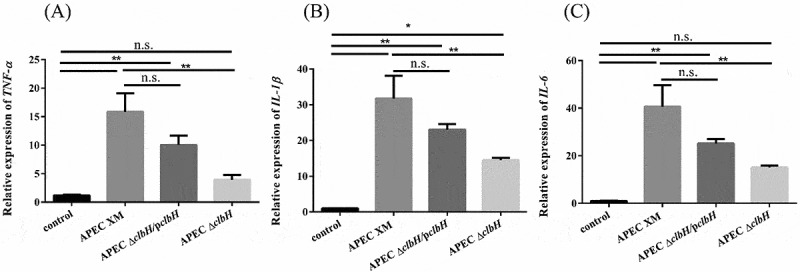
Relative mRNA expression of cytokines in infected bEnd.3 cells

During the development of meningitis, *E. coli* induces an increase in the local production of inflammatory cytokines in endothelial cells [35]. After 4 h of infection, the relative expression of *tumor necrosis factor alpha* (*TNF-α*) (Figure 4 A), *interleukin-1β* (*IL-1β*) (Figure 4 B), and *IL-6* (Figure 4 C) in the APEC Δc*lbH* infection group were significantly decreased, compared with those in the APEC XM group (*p* < 0.01), while the expressions of above cytokines in APEC Δc*lbH*/p*clbH* group were similar with those in APEC XM group.

After 4 h of treatment and three washes with PBS, the relative abundance of *tumor necrosis factor* (*TNF)-α* (A), *interleukin* (*IL)-1β* (B), and *IL-6* (C) mRNA transcripts in bEnd.3 cells were measured using qRT-PCR. The data were analyzed with one‐way ANOVA and expressed as the mean ± standard error of the mean for three independent experiments. (**, *p* < 0.01, *, 0.01 < *p* < 0.05, n.s., *p* > 0.05.)

### mRNA and protein expression of tight junction proteins in infected bEnd.3 cells

**Figure 5.**
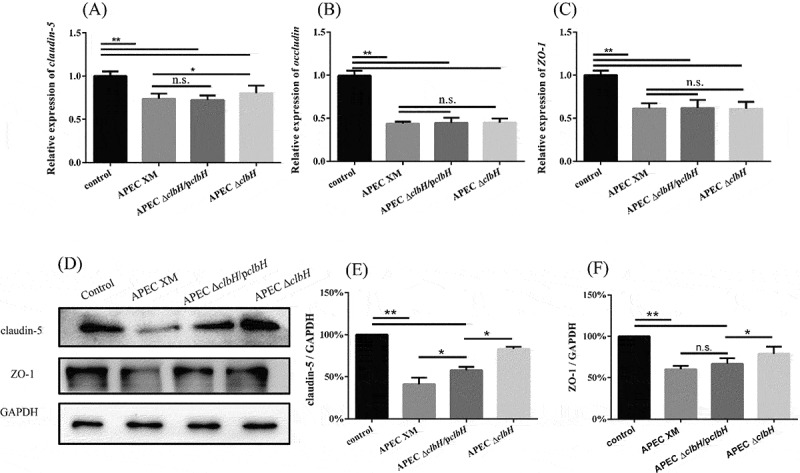
mRNA and protein expression of tight junction proteins in *in vitro* infection

The disruption of tight junction proteins from the BBB is an essential step in meningitis development [36]. The relative expressions of *claudin-5* (*p* < 0.01; Figure 5 A), *occludin* (*p* < 0.01; Figure 5 B), and *ZO-1* (*p* < 0.01; Figure 5 C) in the APEC XM, APEC Δc*lbH*, and APEC Δc*lbH*/p*clbH* groups were significantly decreased compared with those in the control group. The protein expression of tight junction proteins in bEnd.3 cells was also assessed at 4 h post-infection. The expression of ZO-1 and claudin-5 proteins decreased significantly in the APEC XM and APEC Δ*clbH*/p*clbH* groups (*p* < 0.01) compared to that in the control group (Figure 5 D-F). In addition, there were no significant differences in ZO-1 and claudin-5 protein expression between the control and APEC Δ*clbH* groups (*p* > 0.05; Figure 5 D-F).

After 4 h of treatment and three washes with PBS, the relative mRNA expression of *claudin-5* (A), *occludin* (B), and *ZO-1* (C) mRNA transcripts in bEnd.3 cells of the four groups were obtained. *In vitro* Western blot of ZO-1 and claudin-5 (D). Densitometric analysis of claudin-5 (E) and ZO-1 (F) proteins. Protein levels were determined and normalized to those of GAPDH. ZO-1 and claudin-5 proteins decreased significant**l**y in the APEC XM and APEC Δ*clbH*/p*clbH* groups compared to those in the control group. The data were analyzed with one‐way ANOVA and expressed as the mean ± standard error of the mean for three independent experiments. (**, *p* < 0.01, *, 0.01 < *p* < 0.05, n.s., *p* > 0.05.)

### *Colibactin plays an important role in the* in vivo *pathogenicity of APEC XM*

**Figure 6.**
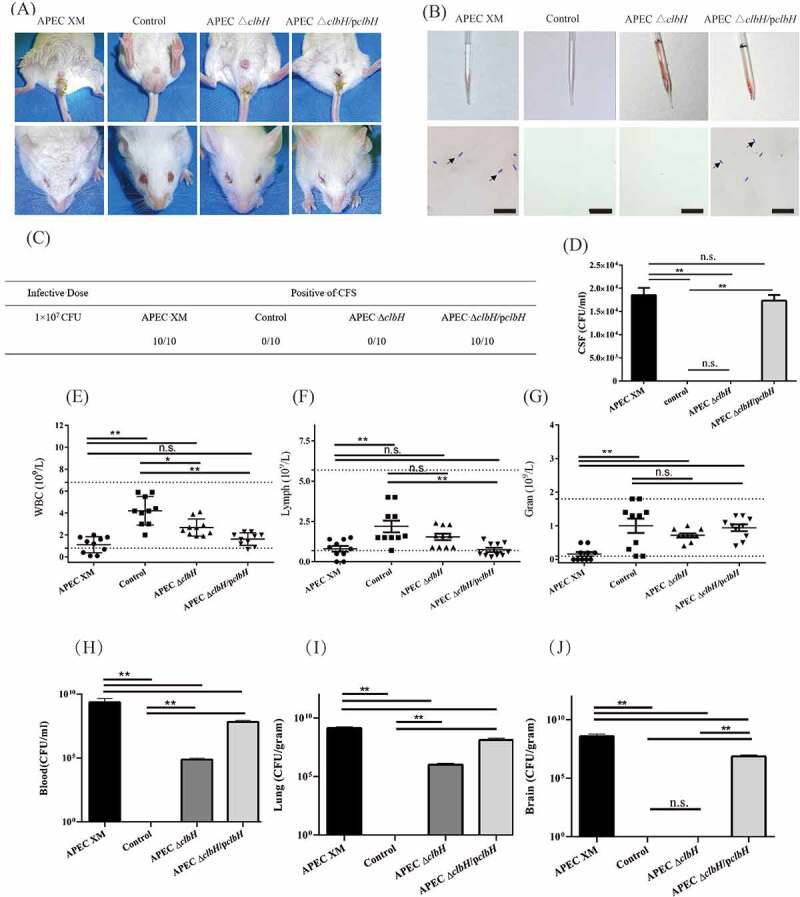
Pathogenicity of APEC XM, APEC Δ*clbH* or APEC Δ*clbH/*p*clbH.*

The mice were monitored continuously, and their clinical symptoms were recorded after 8 h poi. Lethargy, unresponsiveness, lackluster coat, eyelid closure with thick red eye discharge, diarrhea, and neurological symptoms were observed in APEC XM-infected mice (Figure 6A). The mice in the APEC Δ*clbH*/p*clbH* group showed clinical symptoms similar to those described above. In the APEC Δ*clbH* group, the mice presented with mild or no clinical signs (Figure 6A).

Pathogenicity of APECXM, APEC Δ*clbH*, or APEC Δ*clbH/*p*clbH* strains in 4-week-old ICR mice injected intraperitoneally with 10^7^ colony forming units (CFU) of the three strains. The mouse of the control group injected intraperitoneally with an equal volume of sterile saline.(A) Representative images of the mice in each group. The mice in the APEC XM and the APEC XM Δ*clbH/*p*clbH* groups displayed a lackluster coat, diarrhea (upper lane), and eyelid closure with thick red ocular discharge (lower lane). (B) Physical examination (upper lane) and Diff-Quik staining (lower lane) of the cerebrospinal fluid (CSF) samples. *E. coli* (Black arrow) was found in the CSF samples of the APEC XM and APEC XM Δ*clbH/*p*clbH* groups (bar = 10 μm). (C) The presence of *E. coli* in CSF staining is a positive standard for meningitis. (D) Calculation of bacteria from CSF samples. (E-G) Complete blood count (CBC) test data for white blood cells (E), lymphocytes (F), and neutrophils (G) are shown in a scatter plot. Dotted lines in the scatter plot charts represent the normal range of the cell population. The absolute counts of white blood cells, lymphocytes, and neutrophils declined in the APEC XM and APEC Δ*clbH/*p*clbH* groups. (H-J) Bacterial load assay for the blood, lung, and brain, and calculated from blood (CFU/ml) (H), lung (CFU/g) (I), and brain (CFU/g) (J) samples among the four groups determined by plate counting. The data were analyzed with one‐way ANOVA and expressed as the mean ± standard error of the mean for three independent experiments. (**, *p* < 0.01, *, 0.01 < *p* < 0.05, n.s., *p* > 0.05.)

The CSF staining confirmed that all mice in the APEC XM and APEC Δ*clbH*/p*clbH* groups had meningitis (Figure 6 B, C). No mice had meningitis in the APEC Δ*clbH* group (Figure 6 B, C). The bacterial loads in CSF samples from the APEC Δ*clbH*/p*clbH* group were similar to those from the APEC XM group (Figure 6 D). The complete blood count analysis showed that the absolute white blood cell and lymphocyte counts in the APEC XM and APEC Δ*clbH*/p*clbH* groups were lower than those in the control group (*p* < 0.01; Figure 6 D-F). There were no significant differences in absolute neutrophil counts among the APEC Δ*clbH*, APEC Δ*clbH*/p*clbH*, and control groups (*p* > 0.05; Figure 6 E-G).

At 12 h poi, bacterial loads in the lungs and blood significantly decreased in the APEC Δ*clbH* and APEC Δ*clbH*/p*clbH* groups, compared with those in the APEC XM group (*p* < 0.01; Figure 6 H, I). Importantly, no bacteria were isolated from brain tissue samples from the APEC Δ*clbH* or control groups (Figure 6 J). The APEC Δ*clbH*/p*clbH* bacterial load in the brain of mice was significantly reduced compared to that in the APEC XM group (*p* < 0.01; Figure 6 J).

### Relative cytokine mRNA expression, pathological features, and MRI findings in mouse brains

**Figure 7.**
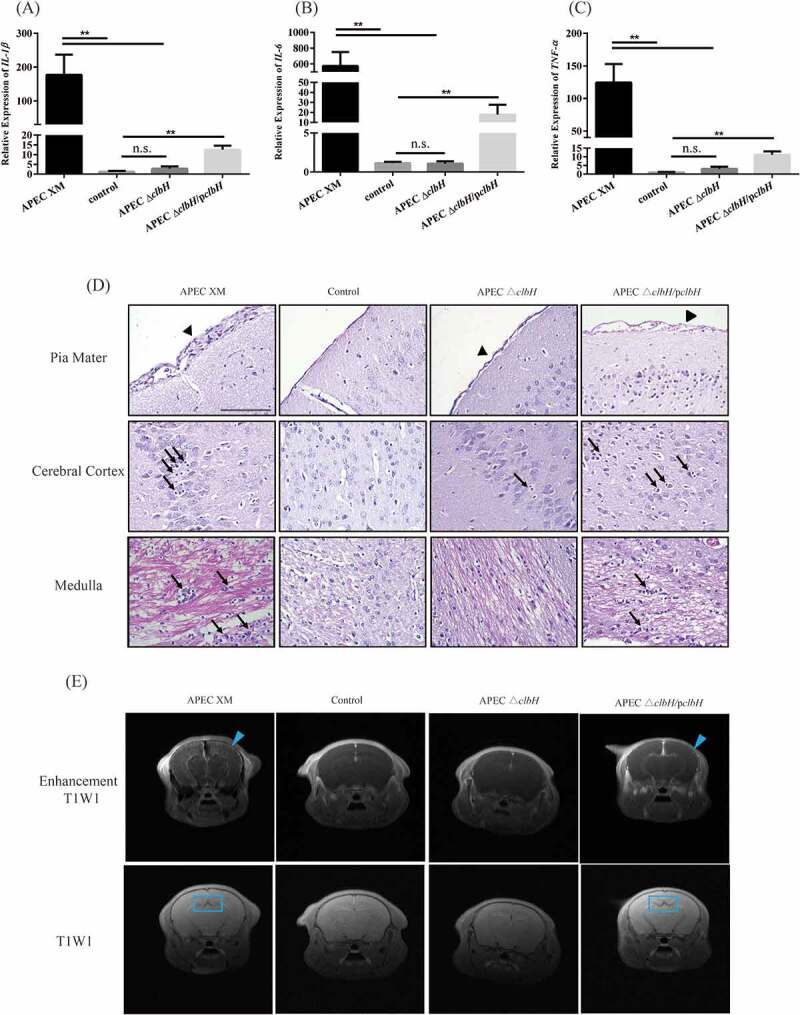
Colibactin contributes to inflammatory responses and brain damage in mice meningitis

The relative expression levels of *IL-1β, IL-6*, and *TNF-α* mRNA in the brain tissue samples were measured by qRT-PCR. In contrast to the control group, the relative expression of all detected cytokines increased significantly (*p* < 0.01; Figure 7 A-C) in the APEC XM group. Compared with that in the APEC XM group, there was a significant decrease (*p* < 0.01; Figure 7 A-C) in the mRNA expression of *IL-1β, IL-6*, and *TNF-α* in the APEC Δ*clbH* group, which was similar to that observed in the control group (*p* > 0.05; Figure 7 A-C). The relative mRNA expression of *IL-1β, IL-6*, and *TNF-α* increased significantly in the APEC Δ*clbH*/p*clbH* group compared with that in the control group (*p* < 0.01; Figure 7 A-C).

4-week-old ICR mice were injected intraperitoneally with 10^7^ colony forming units (CFU) of APEC XM, APEC Δ*clbH*, or APEC Δ*clbH*/p*clbH*. The mouse of the control group injected intraperitoneally with an equal volume of sterile saline. The relative abundance of *IL-1β* (A), *IL-6* (B), and *TNF-α* (C) mRNA transcripts in the brain samples from the four groups. (normalized to *GAPDH*). The results were analyzed with one‐way ANOVA and expressed as the mean ± standard error of the mean for three independent experiments. (**, *p* < 0.01, *, 0.01 < *p* < 0.05, n.s., *p* > 0.05.) (D) Histopathological analysis of the brain tissues. Histopathological features of the pia mater (upper lane), cerebral cortex (middle lane), and medulla (lower lane) in the brain. Leukocyte infiltration in the pia mater, cerebral cortex, and medulla (Black Arrow), thickening of the pia mater (Black Arrowhead, bar = 100 μm). (E) Magnetic resonance imaging (MRI) analyses of mouse brains. Mouse brains were scanned using MRI T1W1 and enhancement T1W1. Abnormal contrast continuous linear enhancement of the pia mater, and diffusion enhancement of the cerebral parenchyma (Blue Arrowhead) and dilatation of the mesencephalic aqueduct (Blue Rectangular Frame) are observed.

Histopathological analysis showed severe thickening of the pia mater and an infiltration of leukocytes in the pia mater, cerebral cortex, and medulla of the mouse brain induced by APEC XM (Figure 7 D). In addition, there were hemorrhages in the pia mater and an infiltration of leukocytes into the pia mater, cerebral cortex, and medulla in the APEC Δ*clbH*/p*clbH* group (Figure 7 D). No similar changes were detected in the mouse brains of the APEC Δ*clbH* or control groups (Figure 7 D).

MRI examination was performed to assess brain damage at 12 h poi. The enhanced T1W1 image demonstrated an abnormal contrast continuous linear enhancement of the pia mater (Figure 7 E, blue arrowhead) and a diffusion enhancing of the cerebral parenchyma in the APEC XM group. These abnormal MRI features partly decreased in the Δ*clbH*/p*clbH* group compared to those in the APEC XM group. In addition, these typical MRI findings for meningitis were not detected in the APEC Δ*clbH* group (Figure 7 E).

### *Deletion of* clbH *reduced* in vivo *BBB disruption*

**Figure 8.**
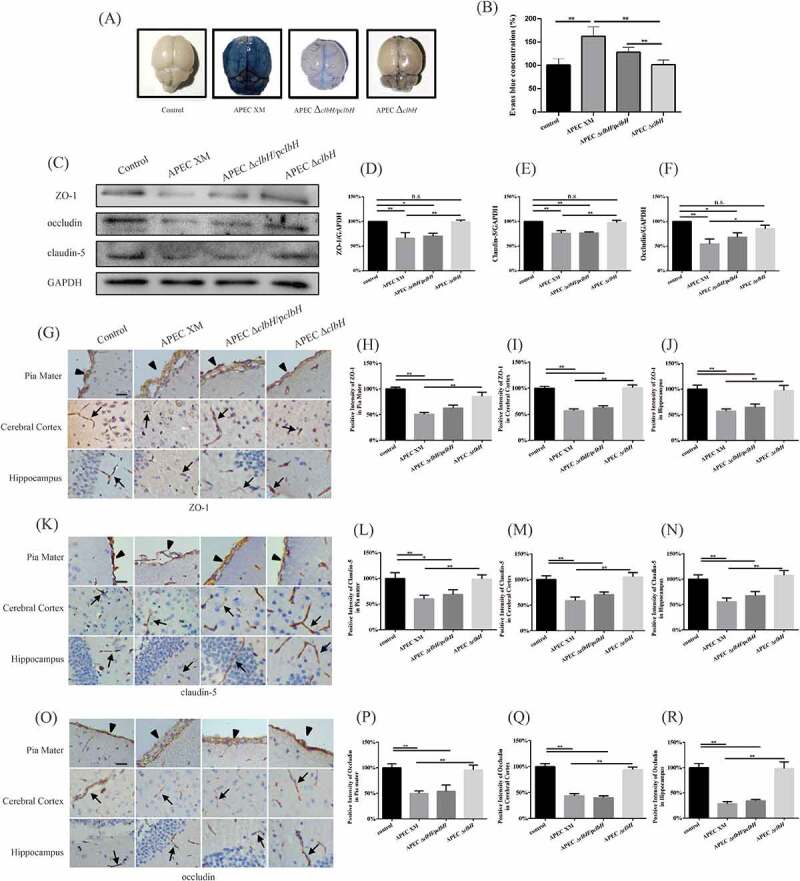
The disruption of blood-brain barrier *in vivo.*

Evans blue staining revealed that the permeability of the BBB increased significantly in the APEC XM group (*p* < 0.01; Figure 8 A, B) compared to that in the control group. The permeability of the BBB decreased significantly in the APEC Δ*clbH* group compared to that in the APEC Δ*clbH*/p*clbH* or APEC XM groups (*p* < 0.01; Figure 8 A, B), which proved that the deletion of *clbH* reduced the disruption of BBB permeability by APEC XM.

*In vivo* disruption of blood-brain barrier (BBB) in 4-week-old ICR mice inoculated intraperitoneally with 10^7^ colony forming units (CFU) of test bacteria for 12 h. The mouse of the control group injected intraperitoneally with an equal volume of sterile saline. (A) The representative bright-field photographs of Evans blue stained mouse brains were taken in dorsal view. (B) Evans blue extravasation from brain homogenates was quantitated at an optical density of 630 nm (OD_630_). The data of the control group was set as 100%. Permeability of the BBB increased significantly in the APEC XM group compared to that in the control group. (C) Representative Western blots of ZO-1, occludin, and claudin-5 *in* *vivo*. Densitometric analysis of ZO-1 (D), claudin-5 (E), and occludin (F) proteins. Protein levels were determined and normalized to those of GAPDH. The data of the control group was set as 100%. ZO-1, occludin, and claudin-5 proteins decreased significantly in the APEC XM and APEC Δ*clbH*/p*clbH* groups, compared to that in the control group. The representative immunohistochemistry staining of ZO-1 (G), claudin-5 (K), and occludin (O) protein levels in the infected mouse brain sections (bar = 20 μm). The black arrowheads point to the pia mater, and the black arrows point to the microvasculature. Immunohistochemistry positive intensity data analysis of ZO-1 protein levels in pia mater (H), cerebral cortex (I) and hippocampus (J); claudin-5 protein levels in the pia mater (L), cerebral cortex (M), hippocampus(N); and occludin protein levels in the pia mater (P), cerebral cortex (Q), and hippocampus (R). The data of the control group was set as 100%. The results were analyzed with one‐way ANOVA and presented as the means ± standard error of the mean for three independent experiments. (**, *p* < 0.01, *, 0.01 < *p* < 0.05, n.s., *p* > 0.05.)

Based on the results above, the changes in tight junctional proteins *in vivo* were also measured by Western blot and immunohistochemical staining. Western blot analysis showed that the protein expression of ZO-1, occludin, and claudin-5 decreased significantly in the brains of mice infected with APEC XM (*p* < 0.01) or APEC Δ*clbH*/p*clbH* (*p* < 0.01) compared with that in the control (Figure 8 C-F). Compared to the APEC XM group, no obvious *in vivo* disruption of ZO-1, occludin, and claudin-5 proteins was found in the APEC Δ*clbH* group (*p* < 0.01; Figure 8 C-F). There were no significant differences between the APEC Δ*clbH* and control groups (*p* > 0.05; Figure 8C-F).

Additionally, immunohistochemical staining confirmed that the expressions of ZO-1, occludin, and claudin-5 in the pia mater, cerebral cortex, and hippocampus of the brain were consistent with those obtained from Western blot (Figure 8 G, K, O). APEC XM and APEC Δ*clbH*/p*clbH* infection resulted in a significant decrease in ZO-1, occludin, and claudin-5 protein expression compared to that in the control group (*p* < 0.01; Figure 8 G, K, O). However, the deletion of *clbH* reduced the ability of APEC XM to disrupt ZO-1, occludin, and claudin-5 expressions. There were no significant differences in these proteins between the APEC Δ*clbH* and control groups (*p* > 0.01; Figure 8 G, K, O).

## Discussion

Despite advances in clinical techniques and antibiotic therapies, *E. coli* meningitis remains a significant cause of mortality [37,38] and neurological disabilities in young infants [39]. Colibactin was first identified in an NMEC strain (IHE3034) [1]. Previous studies have primarily focused on gut homeostasis or colorectal cancer but are limited to neonatal meningitis. In our previous work, we found that the mRNA levels of *pks* island genes, which encode the non-ribosomal peptide synthetase-polyketide synthase (NRPS-PKS) complex for producing colibactin, significantly changed in APEC-XM during infection of bEnd.3 cells [40]. The APEC XM strain was isolated from the brain of a duck with neurological symptoms and septicemia, and the bacteria exhibited meningeal tropism in ICR mice [28,41]. Therefore, a mouse meningitis model was established to elucidate the possible effects of colibactin-induced *E. coli* meningitis.

The NRPS unit on the *pks* island is composed of clbH, clbJ, and clbN [1]. clbH has two N-terminal domains (C-A-T and A1-C-A2-T), and the noncanonical A1 domain activates L-serine to assemble the AM-ACP formation in an analogous manner to the zwittermicin biosynthetic enzymes [23]. Consequently, *pks*^+^ *E. coli* mutants lacking any component of the AM biosynthetic machinery are not genotoxic, and AM-ACP formation is closely related to colibactin assembly [1]. In this study, *clbH* was deleted to construct a non-colibactin-producing strain. Deletion of *clbH* reduced colibactin production and genotoxicity in bEnd.3 cells, which was similar to results obtained from previous studies on *clbA* [42] or *clbP* [16]. As shown in this study, the deletion of *clbH* affected both the cytotoxicity to cells *in vitro* and the pathogenicity to bEnd.3 cells and newborn mice. Many studies have revealed that the successful crossing of the BBB by *E. coli* requires three key steps: a degree of bacteremia, *E. coli* binding to and invasion of brain microvascular endothelial cells, and traversal of the BBB [31–34,43,44]. In this study, we found that deletion of *clbH* did not affect the adhesion and invasion to bEnd.3 cells by APEC XM. Bacterial colonization in organs is concomitant with the capacity to cause bacteremia and systemic infection [5]. Unlike the effective colonization in the APEC XM group, the bacterial loads in the APEC Δ*clbH* group were significantly decreased in the lung, blood, CSF, and brain samples. The mouse serum resistance assay confirmed that APEC Δ*clbH* had lower serum resistance than the APEC XM strain. In line with experimental *pks*^+^ septicemic mice [21], APEC XM and APEC Δ*clbH*/p*clbH* induced profound lymphopenia in meningitis-affected mice, which was alleviated in APEC Δ*clbH*-infected mice. Lymphopenia might reduce the survival rate of mice or humans with sepsis and meningitis induced by *pks*^+^ *E. coli* [21]. Bacterial colonization of the brain is another important step in meningitis development [39]. Brain injuries are a hallmark of meningitis, including necrotic cortical injury and apoptotic hippocampal injury [45,46]. *E. coli* meningitis resembled other bacterial meningitis in the MRI scanning and histopathological characteristics, such as abnormal contrast continuous linear enhancement and severe thickening of the pia mater, leukocyte infiltration into brain tissue, and hemorrhage [47]. However, none of these histopathological and MRI findings were found in the APEC Δ*clbH* group. *clbH* is a necessary component for the synthesis of genotoxic colibactin, which is also strongly associated with meningitis induced by APEC XM.

During the development of meningitis, *E. coli* induces an increase in the local production of inflammatory cytokines in endothelial cells [35], microglia [48], and astrocytes [49]. The expression of *TNF-α, IL-6*, and *IL-1β* in the APEC XM group was in line with that previously reported in *in vivo* or *in vitro E. coli* meningitis studies [50–52]. Overexpression of proinflammatory cytokines could recruit leukocytes into the CNS to create a “cytokine storm” for exaggerated immune responses and CNS damage. In addition, inflammatory mediators play a role in BBB integrity [53,54]. IL-1β contributes to macrophage recruitment, *Streptococcus pneumoniae* clearance [55], and protects mice from lethal gram-negative infections [56]. At the peak of *in vivo* IL-1 expression, there is marked recruitment of neutrophils, breakdown of the BBB, and vasodilatation [57]. High levels of IL-1 in the CSF correlate with the development of neurological complications [58]. TNF-*α* is another important early response cytokine and is related to a fatal outcome in meningitis [58]. Both IL-1β and TNF-α are bone marrow stimulants that grow in a number of myeloid progenitors and promote the recruitment of neutrophils at the inflammation site [58]. TNF-α and IL-1β activate the p38/ERK1/2 pathway and increase myosin light chain kinase [59]. TNF-α also activates the Hif-1α/ VEGF/ VEGFR-2/ ERK signaling pathway to decrease the expression of occludin in human cerebral microvascular endothelial cell lines [60]. IL-6 is a pleiotropic cytokine with both proinflammatory and anti-inflammatory effects. It participates in inflammation, immune response, and hematopoiesis [61] and appears to be a good marker of severity during bacterial infection [62]. IL-6 has emerged as a pivotal player in neuroinflammation due to its influence on the three key branches of this process: astrogliosis [63], microgliosis [64], and BBB integrity. IL-6 increases endothelial permeability and produces ZO-1 mislocalization, actin structure remodeling, and increase in cell contraction [65]. As an anti-inflammatory cytokine in neuroinflammatory conditions, IL-6 maintains BBB integrity by influencing endothelial cells and astrocytes [66]. Inflammatory stress, by using one or a combination of IL-17, IL-6, and/or TNF-α, could lead to the opening of the BBB in the bEnd.3 cell model, which is reflected by a significant increase in permeability and decrease in ZO-1 and claudin-5 [67]. In this study, APEC Δ*clbH* did not evoke *TNF-α, IL-6*, and *IL-1β* in the mouse brain or in bEnd.3 cells, which might have less ability to damage BBB integrity.

The BBB regulates the components in the CNS and minimizes the transfer of toxic compound pathogens to the CNS. BBB disruption is an essential step in meningitis development [36]. The state of brain capillaries and their polarized microvascular endothelial cells is responsible for the BBB structure and functional integrity by possessing tight junctions [68]. Occludin, claudin, junctional adhesion molecules (JAMs), and ZO-1 are the main elements of intercellular tight junction proteins and control the paracellular passage of substrates across the BBB [69]. In this study, we used ZO-1, claudin-5, and occludin to evaluate BBB breakdown. The *in vitro* disruption of ZO-1 and claudin-5 proteins in bEnd.3 cells infected with APEC XM was significantly alleviated in the APEC Δ*clbH* group. In addition, the relative mRNA expression of tight junction proteins in the three strain groups was similar, and decreased significantly compared with that in the control group. In the mouse model, vascular leakage was reduced and tight junction protein breakdown decreased in the APEC Δ*clbH* group. Further, all the tight junction proteins mentioned above decreased at the transcript and/or protein levels in brain endothelial cells in Group B *Streptococcus* [70–72], *Neisseria meningitidis* [73], *Streptococcus suis* [74], or *E. coli* [75] infection. *In vivo*, immunohistochemistry and Western blot assays also showed no significant disruption of tight junction proteins in the APEC Δ*clbH* group. Thus, colibactin might play an important role in disordering tight junction proteins in the BBB. ZO-1 is located on the cytoplasmic surface of endothelial cells. It serves as a recognition protein for tight junction placement and as a support structure for signal transduction [76]. Altering the structure or localization of ZO-1 protein leads to tight junction disconnection, opening of the intercellular gap, and increased BBB permeability. Claudin-5 is present in both human and mouse early fetal brain vessels and continues to increase during postnatal development and maturation of the BBB [77,78]. It is localized specifically to the endothelial cell layer in the brain and is also the most enriched tight junction protein at the BBB [79]. Dysfunction of claudin-5 protein is associated with either neurodegenerative [80], neuroinflammatory [79], or psychiatric disorders [81], and with CNS bacterial infections. Occludin is a central regulatory element in the assembly and function of tight junction proteins, and it is also required for cytokine signal transduction in cells such as for TNF-α and IFNγ [82]. Many studies have demonstrated that changes in redox conditions [83] and interactions with a wide range of kinases and phosphatases [84] can transform occludin domains to disrupt barrier functions.

As it is still difficult to extract pure mature colibactin from bacteria to date [22], it is difficult to investigate the direct role of colibactin on BBB integrity and induction of inflammation in the brain tissue by the tests used in this study. If colibactin can be purified in the future, it could be injected directly into the brain and its pathological effects on the brain tissue may be analyzed. In summary, colibactin is a key virulence factor for APEC XM to induce meningitis. It is responsible for increasing the inflammatory response and decreasing tight junction proteins expression in *in vitro* and *in vivo* infection, which is associated with blood survival mediated by colibactin.

## Data Availability

The datasets generated and/or analyzed during the current study are not publicly available due to the project is not finished yet but are available from the corresponding author on reasonable request.
